# Structural elucidation of triclinic and monoclinic SFCA-III – killing two birds with one stone

**DOI:** 10.1107/S2052520619014380

**Published:** 2019-11-20

**Authors:** Volker Kahlenberg, Hannes Krüger, Valerie Sue Goettgens

**Affiliations:** a Institute of Mineralogy and Petrography, University of Innsbruck, Innrain 52, Innsbruck, A-6020, Austria

**Keywords:** SFCA-III, polysomatism, polytypism, allotwinning

## Abstract

A part of the system CaO-SiO_2_–Al_2_O_3_–Fe_2_O_3_–MgO which is of relevance to iron-ore sintering has been studied in detail. For a bulk composition corresponding to 10.45 wt% CaO, 5.49 wt% MgO, 69.15 wt% Fe_2_O_3_, 13.37 wt% Al_2_O_3_ and 1.55 wt% SiO_2_ synthesis runs have been performed in air in the range between 1100 and 1300°C. Products have been characterized using reflected-light microscopy, electron microprobe analysis and diffraction techniques. At 1250°C, an almost phase-pure material with composition Ca_2.99_Mg_2.67_Fe^3+^_14.58_Fe^2+^_0.77_Al_4.56_Si_0.43_O_36_ has been obtained. The compound corresponds to the first Si-containing representative of the *M*_14+6*n*_O_20+8*n*_ polysomatic series of so-called SFCA phases (**S**ilico-**F**errites of **C**alcium and **A**luminum) with *n* = 2 and is denoted as SFCA-III. Single-crystal diffraction investigations using synchrotron radiation at the X06DA beamline of the Swiss Light Source revealed that the chemically homogenous sample contained both a triclinic and monoclinic polytype. Basic crystallographic data are as follows: triclinic form: *a* = 10.3279 (2) Å, *b* = 10.4340 (2) Å, *c* = 14.3794 (2) Å, α = 93.4888 (12)°, β = 107.3209 (14)° and γ = 109.6626 (14)°, *V* = 1370.49 (5) Å^3^, *Z* = 2, space group ; monoclinic form: *a* = 10.3277 (2) Å, *b* = 27.0134 (4) Å, *c* = 10.4344 (2) Å, β = 109.668 (2)°, *V* = 2741.22 (9) Å^3^, *Z* = 4, space group *P*2_1_/*n*. Structure determination of both modifications was successful using diffraction data from the same allotwinned crystal. A description of the observed polytypism within the framework of OD-theory is presented. Triclinic and monoclinic SFCA-III actually correspond to the two possible maximum degree of order structures based on OD-layers containing three spinel (S) and one pyroxene (P) modules (〈S_3_P〉). The existence of SFCA-III in industrial iron-ore sinters has yet to be confirmed. Polytypism is likely to occur in other SFCA-members (SFCA, SFCA-I) relevant to sintering as well, but has so far been neglected in the characterization of industrial samples. Our results shed light on this phenomenon and may therefore be also helpful for better interpretation of the powder diffraction patterns that are used for phase analysis of iron-ore sinters.

## Abstract 2

Synthesis experiments in the system CaO–SiO_2_–Al_2_O_3_–Fe_2_O_3_–MgO resulting in the formation of SFCA-III are reported. SFCA-III is a new *n* = 2 member of a polysomatic series of *M*_14+6*n*_O_20+8*n*_ compounds based on pyroxene-spinel modules which are of relevance to iron-ore sintering. Single-crystal diffraction studies using synchrotron radiation revealed that the compound occurs in two polytypes representing maximum degree of order structures which explains the observed allotwinning of the sample.

## 1. Introduction   

**S**ilico-**F**errites of **C**alcium and **A**luminum compounds (so-called SFCA’s) are major constituents of iron-ore sinters. Sintering is an important step in the iron-producing process, where loose iron ore fines (< 6 mm) are transformed at temperatures between 1250 and 1350°C into a mechanically stable composite that can be used as a feedstock for the blast furnace (Lu & Ishiyama, 2015 ▸). In the European Union, about 130 million tons of ore have been recently sintered per annum (Fernández-González *et al.*, 2017 ▸), making iron-ore sinters one of the most produced inorganic materials. During the sinter process a moving strand is continuously charged with a mixture of ore (sinter feed), fine coke or anthracite (fuel), limestone (flux), other solid additives as well as water. The charge on the strand is ignited by burners using natural or coke oven gas. After a short ignition time a narrow combustion zone (flame front) is sucked downwards through the bed. In a series of high-temperature reactions a semi-molten porous material – the sinter – is formed including the so-called SFCA phases representing the polycrystalline bonding agent between the different particles (Lu & Ishiyama, 2015 ▸).

Concerning their chemical compositions, different types of SFCA compounds can be distinguished. They represent complex solid solutions corresponding to the general formula *M*_14+6*n*_O_20+8*n*_, where *M* = Si, Fe, Al, Ca and Mg (Hamilton *et al.*, 1989 ▸; Mumme *et al.*, 1998 ▸; Sugiyama *et al.*, 2005 ▸; Liles *et al.*, 2016 ▸; Zöll *et al.*, 2017 ▸; Nicol *et al.*, 2018 ▸). The two most frequently observed representatives in industrial sinters are named SFCA (*n* = 0 or *M*_14_O_20_) and SFCA-I (*n* = 1 or *M*_20_O_28_) (Webster *et al.*, 2012 ▸). Both types may be also distinguished by the morphology of their crystals: Whereas SFCA-I adapts a platy or acicular habit, SFCA forms more columnar-shaped crystals (Webster *et al.*, 2014 ▸). Recent investigations from our group showed, that a ‘silicon-free’ so-called FCAM-III phase with overall stoichiometry *M*_26_O_36_ (*n* = 2) exists as well (Zöll *et al.*, 2018 ▸). The SFCA-types with *n* = 0, 1 and 2 are structurally related forming a polysomatic series, a term that has been coined by Thompson (1970 ▸, 1978 ▸). Actually, the three members can be described by the same approach of stacking sequences of pyroxene (P) and spinel (S) modules or slabs. More details on this relationship will be presented in the *Discussion* [Sec sec4] of this contribution.

Furthermore, there exists another SFCA phase called SFCA-II with *M*_17_O_24_ stoichiometry. *Sensu stricto*, SFCA-II is *not* a direct member of the abovementioned series but represents an intermediate structure type between SFCA and SFCA-I (Mumme, 2003 ▸; Mumme & Gable, 2018 ▸). SFCA-II has been claimed to occur in South African sinters (Van den Berg & deVilliers, 2009 ▸).

Notably, there is a slight inconsistency in nomenclature among the SFCA-phases that might provoke confusion. With the name SFCA-II already in use, Zöll *et al.* (2018 ▸) finally decided to denominate their novel *n* = 2 compound with the Roman numeral ‘III’.

The present contribution reports the results of synthesis experiments in the system CaO-SiO_2_–Al_2_O_3_–Fe_2_O_3_–MgO, the existence of the first ‘real’ (*i.e.* Si-containing) SFCA-III member, its polytypism involving two polytypes of maximum degree of order (MDO) and the observed allotwinning.

## 2. Experimental details   

According to our preliminary studies on the quinary oxide system we focused on samples with a bulk chemistry of 10.45 wt% CaO, 5.49 wt% MgO, 69.15 wt% Fe_2_O_3_, 13.37 wt% Al_2_O_3_ and 1.55 wt% SiO_2_. Starting materials for a total of 3 g were Fe_2_O_3_ (> 99.997%, Alfa Aesar), γ-Al_2_O_3_ (99.997%, Alfa Aesar), MgO (99,998% Alfa Aesar), CaCO_3_ (99.995% Merck) and amorphous SiO_2_ (99.99%, Alfa Aesar). The reagents were dried at 400°C for 24 h and checked for impurities using X-ray powder diffraction (XRPD). Before weighing on an analytical balance, the educts were stored at 110°C in a drying cabinet. A planetary mill operated at 600 rpm was used for homogenization for 45 min under ethanol. The resulting slurry was dried for one day at 50°C to remove the alcohol completely, manually re-homogenized in an agate mortar for 30 min and finally transferred to a desiccator. Before high-temperature treatment, about 0.6–0.8 g of the educts were pressed into pellets each having a diameter of 12 mm and a thickness of about 2 mm. Firing was performed in a resistance heated furnace with an external S-type thermocouple placed next to the open platinum crucibles containing the pellets. The samples were heated from 300°C (with a ramp of 90°C h^−1^) to about 100°C below the respective maximum temperatures (1100, 1200, 1250 and 1300°C). For the last 100°C, a slower rate of 30°C h^−1^ was employed to avoid over-heating of the sample. After a total experimental time of 72 h the samples were immediately quenched in a water bath. Weight losses were determined from weight differences before and after heating.

XRPD data have been acquired with a Stoe Multi-Purpose Diffractometer system in horizontal θ–2θ Bragg–Brentano geometry in combination with a one-dimensional Mythen 1K detector. The device is equipped with a primary-beam Ge(111) monochromator yielding *K*α_1_-radiation only. In order to avoid fluorescence problems due to the iron containing samples the diffraction studies were performed with a cobalt tube operated at 40 kV and 30 mA. Data were collected at ambient temperature over a 2θ range between 2° and 120° and a step size of 0.015° 2θ. The total measurement time per sample was about 48 h. Evaluation of the phase content was performed with the 2018 release of the PDF-4+ Powder Diffraction File database of the International Centre for Diffraction Data (ICDD, 2018 ▸). For LeBail fits of the patterns of the crystalline synthesis products the program *FullProf.2k* version 3.3 (Rodriguez-Carvajal, 2005 ▸) was used. Thompson–Cox–Hastings pseudo-Voigt functions (Thompson *et al.*, 1987 ▸) were chosen for the simulation of the peak shape. Furthermore, an asymmetry correction following Finger *et al.* (1994 ▸) was included. For simulation of the background*,* a six-coefficient polynomial was selected.

To obtain the chemical compositions of the observed phases and to visualize the phase relations in the experiments, parts of the synthesized material were embedded in ep­oxy resin, polished with diamond pastes of different grain sizes varying from 10 to 1 µm and finally sputter-coated with gold. Electron microprobe analysis (EMPA) in wavelength-dispersive mode was performed using a Jeol JXA SUPERPROBE 8100. Measurements were accomplished with an acceleration voltage of 15 kV, a beam current of 10 nA and counting times of 20 s (on peaks) and 10 s (for background on each side of the peaks), respectively. Depending on grain size, between 1 and 25 spots in various regions of the samples were analyzed. The standard reference materials used for the analysis were quartz (Si), diopside (Ca, Mg), corundum (Al) and magnetite (Fe). In addition to point analyses with a focal spot diameter of 2 µm, for one sample an area of 120 µm × 120 µm was mapped. Intensities were corrected for electron scattering, absorption and fluorescence radiation (so-called ZAF-correction).

Finally, the Fe^2+^/Fe_tot_ ratio was calculated from the total iron content based on the crystal-chemical formula obtained from EMPA measurements and charge balance considerations.

Preliminary single-crystal diffraction studies were performed on a Rigaku Oxford Diffraction Gemini R Ultra single-crystal diffractometer (equipped with a Ruby CCD detector) using both Mo *K*α and Cu *K*α radiation. Therefore, parts of the sinter pellets were crushed in an agate mortar and further checked using a polarizing binocular. Fragments were mounted in plastic cryoloops (*Litholoops*, Molecular Dimensions) using an inert oil and cooled in a −100°C dried air stream generated by an Oxford Cryosystems Desktop Cooler. Unfortunately, the 20–40 µm sized samples were diffracting only very weakly despite long exposure times for both molybdenum and copper radiation, respectively. Therefore, we finally decided to re-mount the most promising samples on thin glass fibers with a two-component adhesive and to collect single-crystal diffraction data at the X06DA beamline of the Swiss Light Source, Paul Scherrer Institute, Villigen, Switzerland. The usage of this synchrotron beamline offers not only a much higher brilliance of the beam but also provides a Pilatus 2M-F detector (Dectris Ltd, Baden-Daettwill, Switzerland) with a sharp point-spread function. This experimental setup is a definite advantage when dealing with intergrown samples and turned out to be crucial for the final success of this study. Diffraction experiments were performed at 22°C using the DA+ acquisition software (Wojdyla *et al.*, 2018 ▸). The wavelength was tuned to 0.72931 Å. The detector was placed 80 mm from the sample resulting in a maximum resolution of 0.7 Å. One thousand eight hundred frames were recorded using fine-sliced (0.2°) ω-scans with 0.1 seconds per frame. Table 1 ▸ contains a summary of conditions pertaining to the specific data collection of the sample that was used for final structure elucidations. The *CrysAlis Pro* software package (Rigaku Oxford Diffraction, 2015 ▸) was employed for indexing, integration and data reduction including Lorentz and polarization as well as an empirical absorption correction. For structure solution and subsequent least-squares refinements the program suite *JANA2006* (Petřiček *et al.*, 2014 ▸) was used. X-ray scattering factors for neutral atoms together with real and imaginary coefficients for anomalous dispersion were taken from Volume C of the *International Tables for Crystallography* (2004 ▸). A summary of the relevant basic crystallographic data can be found in Table 2 ▸. Figures showing structural details were prepared with the program *VESTA* (Momma & Izumi, 2008 ▸).

## 3. Results   

### 3.1. Synthesis   

The pellet quenched from 1100°C had a brownish color and exhibited no remarkable shrinkage. Reflected-light microscopy (50× magnification) showed bright clear gray crystals embedded in a fine-grained reddish matrix. Electron microprobe analysis (EPMA) measurements revealed that quartz, hematite, melilite, SFCA-I and a further phase that probably represents Ca-poor FCAM-III are present (see Table 3 ▸). The first two compounds correspond to the starting reagents indicating that the high-temperature reactions were not complete. Notably, reliable chemical analysis using the micro-probe was hindered by the substantial porosity and the small crystallite sizes of the sample.

The pellet fired at 1200°C showed a distinct volume reduction and a black color. In contrast to the 1100°C experiment the tablet was significantly harder when crushed in an agate mortar. Intensively intergrown subhedral crystals up to 50 µm in size could be observed. From the EPMA measurements two crystalline phases could be identified. Most of the sample consisted of a Si-containing equivalent of FCAM-III, which will hereinafter be referred to as ‘SFCA-III’. Furthermore, small amounts of melilite could be detected (see Table 3 ▸).

**Figure 1:**
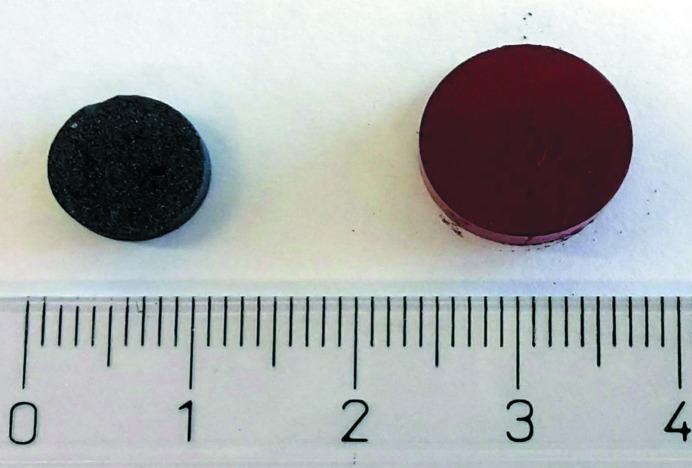
Visual comparison between the pellets after (left) and before (right) sintering at 1250°C: a distinct change in color and a pronounced shrinkage is evident.

**Figure 2:**
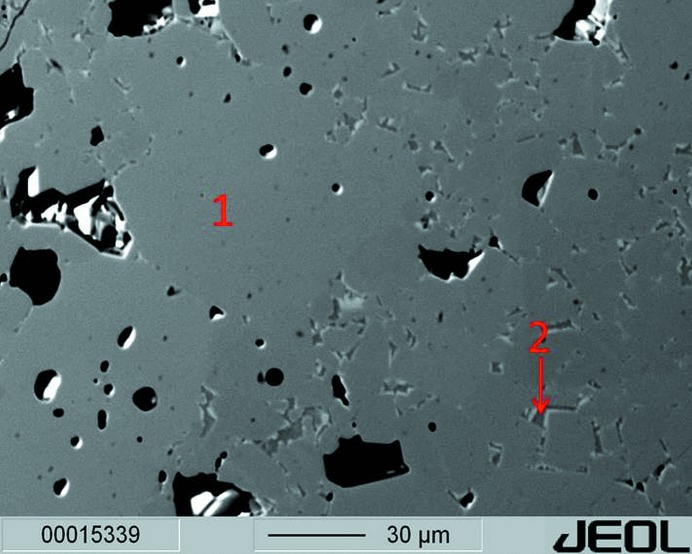
BSE-image of the sample from the 1250°C experiment. The predominant portion (1) of the sample corresponds to chemically homogeneous SFCA-III. Furthermore, chemically different interstitial vugs (2) can be identified (see text).

Increasing the reaction temperature to 1250°C triggered an even more pronounced sintering shrinkage (see Fig. 1 ▸). With the naked eye the black pellet looked homogeneous. In comparison with the 1200°C run, reflected-light microscopy revealed slightly larger and less-intergrown crystals. Well developed faces and sometimes even idiomorphic shapes could be observed. Again, an increase in hardness was detected. Chemical analysis proved the presence of two phases. The predominant portion of the sample corresponded to chemically homogeneous SFCA-III showing no spatial variation in stoichiometry. Its composition is very close to the one observed at 1200°C. Furthermore, chemically different interstitial vugs have been identified (see Table 3 ▸ and Fig. 2 ▸). However, due to their small size we cannot exclude the possibility that their composition could represent the result of mixing analyses.

At 1300°C partial melting was observed. The black pellet had softened, sticked to the bottom of the crucible and had to be removed mechanically. By means of reflected-light microscopy idiomorphically developed crystals up to 50 µm in size could be identified, which were embedded in a polycrystalline homogeneous matrix. Observed colors were light gray or almost black. The lighter crystals corresponded to SFCA-III, whereas the darker phase could be attributed to SFCA-I. According to the backscattered electron (BSE) images and the EMPA measurements there are indications that many of the SFCA-I grains showed an Mg- and Al-enriched core and a very thin Fe- and Ca-enriched rim (see Table 3 ▸). Again, an influence of mixing analyses on the composition of these peripheral areas cannot be excluded. The inter-granular matrix had a different virtually Si-free composition and probably represents the previous melt phase.

### 3.2. X-ray single-crystal diffraction and structure refinement   

Diffraction data of several samples from the 1250°C synthesis run were acquired using synchrotron radiation at the Swiss Light Source. Two of those (named A and B) showed the best diffraction quality and were studied in more detail. The single-crystal diffraction pattern of crystal A could be completely indexed using a triclinic unit cell close to the one reported by Zöll *et al.* (2018 ▸) for FCAM-III. Subsequent refinement calculations in space group 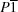 based on the model of Zöll *et al.* (2018 ▸) actually confirmed an isostructural relationship between the Si-containing crystal A and Si-free FCAM-III. In contrast, the diffraction pattern of crystal B exhibited a much higher level of complexity. At first glance, the automatic unit-cell finding algorithm indicated the presence of a triclinic cell similar to the one observed for crystal A with the following lattice parameters: *a* = 10.3279 (2) Å, *b* = 10.4340 (2) Å, *c* = 14.3794 (2) Å, α = 93.4888 (12)°, β = 107.3209 (14)° and γ = 109.6626 (14)°. A detailed analysis of the diffraction data involving precession-type reconstructions of reciprocal space, however, showed that not all observed reflections could be successfully indexed with this cell. Various kinds of superstructures or non-merohedral twinning of the triclinic cell were considered as explanations. Actually, this type of twinning has been already reported to occur for other members of the SFCA polysomatic series (Walenta, 1969 ▸; Cosca *et al.*, 1988 ▸; Zöll *et al.*, 2018 ▸). However, neither of the two potential reasons could resolve the problem. Finally, we came up with a solution where a second monoclinic cell had to be introduced in order to interpret the diffraction pattern completely. So far the corresponding monoclinic metric has not been described among the different SFCA representatives: *a* = 10.3277 (2) Å, *b* = 27.0134 (4) Å, *c* = 10.4344 (2) Å, β = 109.668 (2)°. Notably, both observed reciprocal lattices show a strict orientational relationship that can be expressed by the following equations between the reciprocal lattice vectors a*, b* and c*:
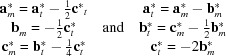



The resulting relationships between the indices of the reflections are as follows: *h_t_* = *h_m_*; *k_t_* = *l_m_*; *l_t_* = − [(*h_m_* + *k_m_*)/2 + *l_m_*/4]. Therefore, the superposition of the respective reciprocal lattices results in different subsets of either completely overlapping or completely separated reflections. Overlapping occurs for reflections with (*h_m_* + *k_m_*) = 2*n* if *l_m_* = 4*n* or (*h_m_* + *k_m_*) = 2n+1 if *l_m_* = 4*n*+2.

**Figure 3:**
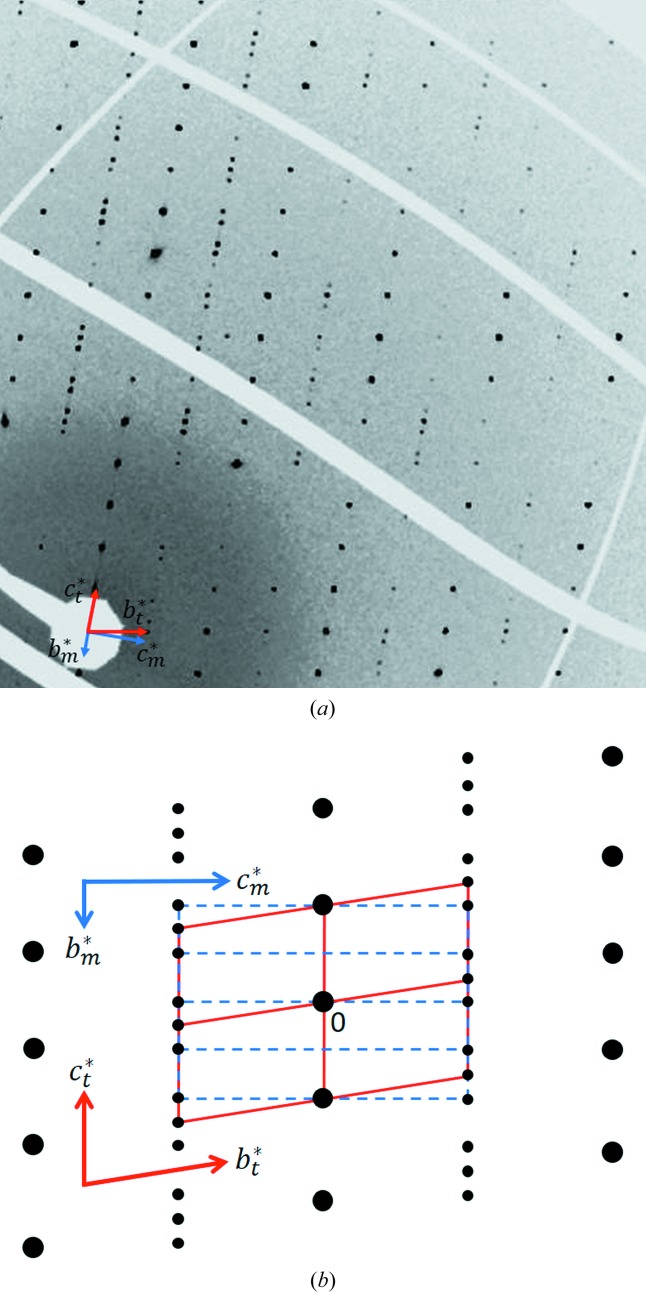
(*a*) Reconstructed part of the (0*kl*)_mon_ layer of reciprocal space of the sample B. (*b*) Schematic sketch explaining the observed pattern of the layer as a superposition of two sets of reciprocal lattice vectors.

The superposition is exemplarily shown in Fig. 3 ▸ for the (0*kl*)-layer. From the abovementioned equations the following dependencies between the lattice vectors ***a*, *b*** and ***c*** in direct space have been derived:
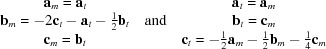



The analysis of the systematic absences for the monoclinic component indicated space group *P*2_1_/*n*. In summary one can say, that the apparent ‘single-crystal’ B actually represents a multi-crystal with an oriented intergrowth of two *different* crystalline phases.

Structure determination of the monoclinic phase proceeded as follows. First, two separate integrations based on the triclinic and the monoclinic cells were performed. Naturally, each of these data sets also contained overlapping reflections that were systematically biased due to intensity contributions coming from the other phase. Using the model determined from crystal A as a starting point for the refinement of the triclinic component of the multi-crystal B resulted in a weighted residual *wR*(*F* ^2^) = 0.0859. A first structure model of the monoclinic component was accomplished by applying the charge flipping method (Oszlányi & Sütő, 2004 ▸) implemented in the program *SUPERFLIP* (Palatinus & Chapuis, 2007 ▸). In the next step, the two aforementioned data sets were simultaneously utilized for the least-squares optimization of the structure parameters of both phases (‘multi-crystal option’ of *JANA2006*). The previously determined transformation matrix between the triclinic and monoclinic lattices allowed a classification of the total 39 442 collected intensities into 26 137 ‘pure monoclinic’ and 13 302 ‘pure triclinic’ reflections. Overlapping reflections accounted for 6534 diffraction spots. The high-quality of the synchrotron diffraction data enabled the refinement of the fractional atomic coordinates and anisotropic displacement parameters for *all* atoms of both phases. The final calculations converged to a residual of *wR*(*F* ^2^) = 0.037 for 1170 parameters and 14 341 reflections with *I* > 2σ(*I*) (Table 1 ▸). The resulting volume fractions of the monoclinic and triclinic phase within sample B were determined as 62.5 (7)% and 37.5 (7)%, respectively.

The analysis of the distributions of the different cation species on the different tetrahedral and octahedral positions present in both phases was complicated by the facts that (i) the assignment of more than two chemical species to a specific site is mathematically undetermined and (ii) Mg, Al and Si have almost identical scattering factors for X-rays. Therefore, for the site-occupancy investigations the following procedure has been applied: Initially, the magnesium and silicon contents were completely neglected and the total scattering power for each site was obtained from the structure refinements by allowing for a potential Fe ↔ Al substitution under the assumption of full occupancy for each position. The assignment of different chemical species (Fe, Al, Mg, Si, Ca) to each site was performed *a posteriori* using the program *OccQP* (Wright *et al.*, 2000 ▸). The software tries to find the ‘optimal’ solution concerning cation distributions by simultaneously minimizing the differences between observed and calculated values for (i) chemical composition, (ii) total site scattering values (as determined from single-crystal structure analysis), (iii) bond-valence sums and (iv) bond lengths. Due to the chemically homogenous character of the bulk sample observed in the BSE images from the relevant 1250°C synthesis we decided to use the same stoichiometry from the EPMA measurements for both phases: Ca_2.99_Mg_2.67_Fe^3+^_14.58_Fe^2+^_0.77_Al_4.56_Si_0.43_O_36_. This composition was introduced as an additional constraint during the optimization. For the terms (ii)–(iv) equal unit weights were used. Furthermore, additional crystallochemical input has been employed (Si-avoidance on the octahedral and Ca-avoidance on the tetrahedral sites). As far as possible, the labels of the T and M positions have been chosen to facilitate a comparison between their location in the layer-like building blocks of the triclinic and the monoclinic polymorph (see *Discussion* [Sec sec4]).

### 3.3. X-ray powder diffraction   

**Figure 4:**
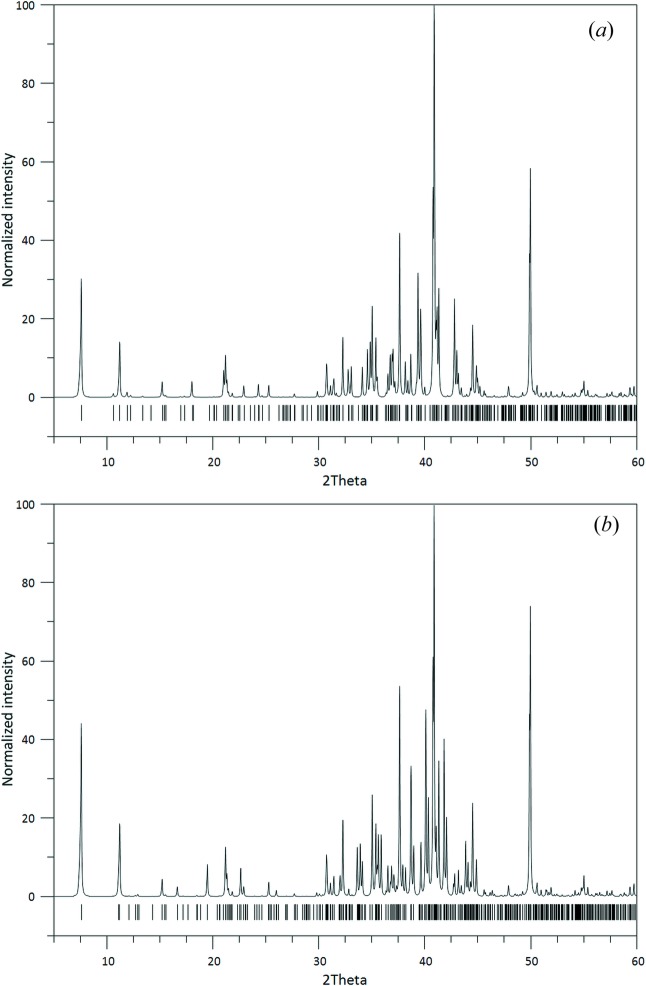
Calculated X-ray powder diffraction patterns (Co *K*α_1_ radiation) for (*a*) triclinic and (*b*) monoclinic SFCA-III based on the structural models determined in this publication. The strong similarities are evident.

Calculated powder diffraction data based on the determined structures of triclinic and monoclinic SFCA-III are presented in Fig. 4 ▸(*a*) and (Co *K*α_1_ radiation). Principally, the powder diffractograms of both modifications show a large number of coinciding reflections with comparable intensities, making a straightforward differentiation by X-ray powder diffraction difficult. This feature is characteristic for polytypic families where intense common (so-called family reflections) can be distinguished from weak so-called characteristic reflections carrying the information about the differences between the members of the family (Merlino, 1997 ▸). The figures suggest that the most promising region to discriminate the two polymorphs is the 2θ range 15–25°.

**Figure 5:**
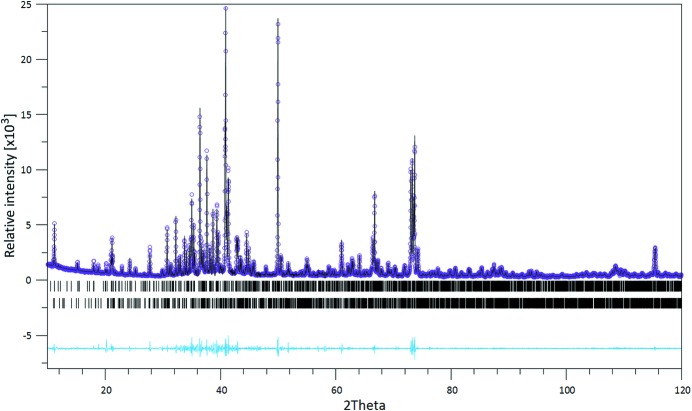
LeBail-fit of the powder pattern acquired for the sample prepared at 1250°C (Co *K*α_1_ radiation) at ambient conditions. Observed step intensities are represented by small circles. Calculated step intensities (solid line) have been modeled based on a mixture of the two polytypes of SFCA-III. Tick marks for the Bragg peaks of each phase are given (first row: triclinic SFCA-III; second row: monoclinic SFCA-III). The lower line represents the difference curve between observed and calculated step intensities. 2θ given in °.

Fig. 5 ▸ shows a LeBail-fit of the data acquired from the polycrystalline sample synthesized at 1250°C. Fitting was based on the assumption of the simultaneous presence of both polymorphs. The resulting profile residuals were *R*_p_ = 0.081 and *R*_wp_ = 0.114, respectively. The first observed peak at about 7.5° 2θ had to be excluded due to its strong asymmetry that could not be modeled adequately. Calculations based on the existence of only one polymorph resulted in instable refinements and considerably worse residuals. The resulting lattice parameters from the two-phase fit are as follows: *a* = 10.33293 (6) Å, *b* = 27.0360 (1) Å, *c* = 10.42690 (7) Å, β = 109.7953 (5)° (monoclinic SFCA-III) and *a* = 10.31334 (4) Å, *b* = 10.42257 (5) Å, *c* = 14.35999 (5) Å, α = 93.5169 (4)°, β = 107.3449 (4)° and γ = 109.5948 (3)° (for triclinic SFCA-III). These values compare reasonably well with the ones obtained in the single-crystal diffraction study. At any rate, the standard uncertainties of the lattice parameters are significantly higher than those listed above, which have been obtained from the Rietveld analysis software and reflect only the precision of the mathematical fit between measured and calculated step intensities.

## 4. Discussion   

The crystal structures of triclinic and monoclinic SFCA-III are closely related. For their description we will start with their common features. The differences between them will be addressed later on in this section.

**Figure 6:**
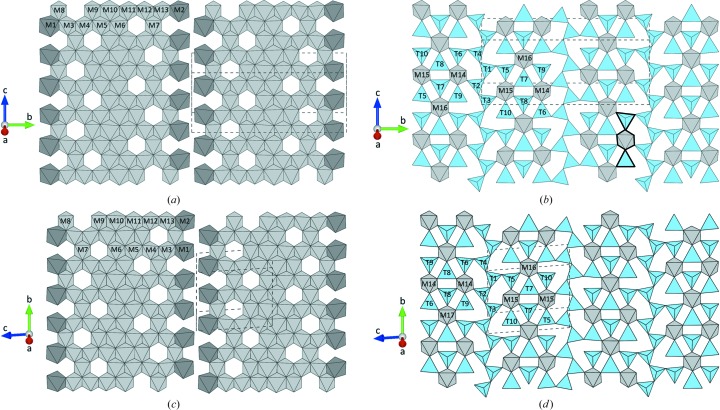
Principal layers observed in the SFCA-III modifications containing band-like units of octahedra (layer 1) as well as so-called winged-octahedra and *vierer*-single chains of tetrahedra (layer 2). Layers are presented in projections perpendicular to the sheets. (*a*) and (*b*): monoclinic form; (*c*) and (*d*): triclinic form. Dark-gray octahedra represent pure [CaO_6_]-units, whereas the octahedra about the remaining M-sites are given in light-gray. Tetrahedra are marked in blue. For sake of clarity, the contour of a single winged-octahedron is highlighted with bold lines.

The two SFCA-III modifications are based on an alternating sequence of two different types of layers. Layer type 1 consists of band-like structures [see Figs. 6 ▸(*a*) and 6 ▸(*c*)]. Within a single band the MO_6_-octahedra share common edges. Notably, not all of the potentially available octahedral sites are actually occupied resulting in the formation of ordered vacancies. Furthermore, there is no direct linkage between adjacent bands within a single layer of type 1. For both SFCA-III modifications the layers of type 1 contain inversion centers. Layer type 2 contains (i) heteropolyhedral units comprising an MO_6_-octahedron sharing two corners with neighboring [TO_4_]-units (so-called ‘winged octahedra’) as well as TO_4_-tetrahedra forming *vierer* single-chains (T) [see Figs. 6 ▸(*b*) and 6 ▸(*d*)]. In more detail, the winged octahedra (Mumme, 2003 ▸) represent MT_2_O_12_-clusters, where the central MO_6_-moieties share *trans*-vertices with the adjacent two tetrahedra. These clusters in turn are arranged in ribbons (W) containing three of these units, *i.e.* a single sheet of type 2 can be constructed from a succession of linear building elements forming a …T-3W-T-3W-… sequence. Layers of type 2 are slightly different in the triclinic and monoclinic modifications of SFCA-III since they differ in their local symmetry elements. While the triclinic form contains inversion centers (as in layer type 1), the type 2 layers in the monoclinic phase comprise 2_1_-screw axes parallel [010]. Furthermore, two of the MO_6_-polyhedra about M16 and M17 in layer type 2 of the triclinic modification reside on special positions with site symmetry 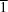.

**Figure 7:**
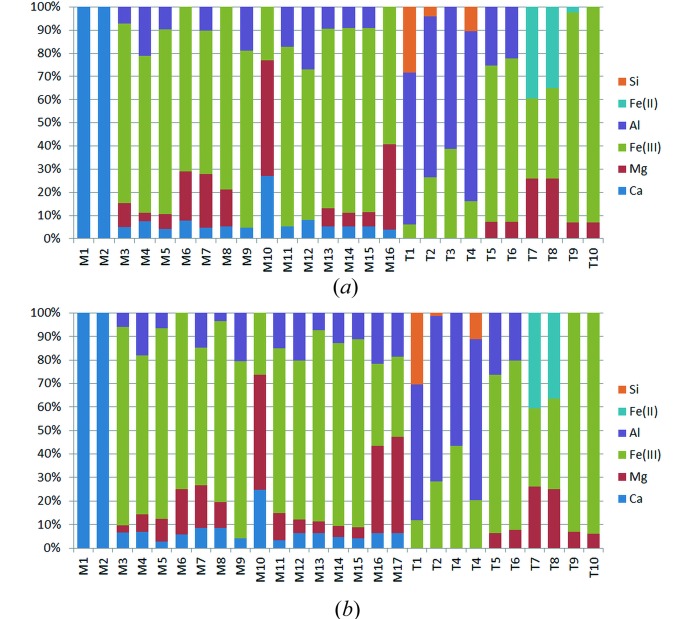
Bar graphs for (*a*) monoclinic and (*b*) triclinic SFCA-III visualizing the cation distributions among the different M- and T-sites.

The cation distributions on the M- and T-sites as obtained from the aforementioned *OccQp* calculations are summarized Figs. 7 ▸(*a*) and 7 ▸(*b*). The distributions among the corresponding sites in the two polymorphs are not completely identical but exhibit a high degree of similarity. For example, the M1 and M2 polyhedra located at the rims of the octahedral bands are exclusively occupied by calcium cations. The remaining Ca ions are distributed among the remaining M-positions with a preference for the M10-site in the very center of the bands. Al occurs on both the M- and T-sites. However, the positions T1, T2, T3 and T4 within the *vierer* single-chains host the largest amounts of aluminium. Fe^II^ is concentrated on the T7 and T8 sites, whereas larger quantities Fe^III^ can be found in the centers of all polyhedra except for T1 and the two pure calcium sites M1 and M2. Finally, magnesium is enriched on M10, M16/M17, T7 and T8, respectively.

A different understanding of SFCA-III can be obtained when comparing it with the so-called pyroxene-spinel family of polysomatic structures (Zvyagin & Merlino, 2003 ▸). This group of compounds is built from two different structural modules which represent layers that can be imagined as being cut from the well known pyroxene (P) and spinel (S) structure-types. Actually, these layers are more or less perpendicular to the sheets of type 1 and 2 that have been mentioned above. A large number of minerals from the sapphirine-aenigmatite group (Bonaccorsi *et al.*, 1990 ▸; Shchipalkina *et al.*, 2016 ▸; Galuskina *et al.*, 2017 ▸), meteoritic Ca_2_Al_12_O_20_ (Ma *et al.*, 2017 ▸) as well as the synthetic compounds such as (Ge_2_Mg_4_Ga_8_)O_20_ (Barbier, 1990 ▸) or the SFCA-series can be attributed to this family.

**Figure 8:**
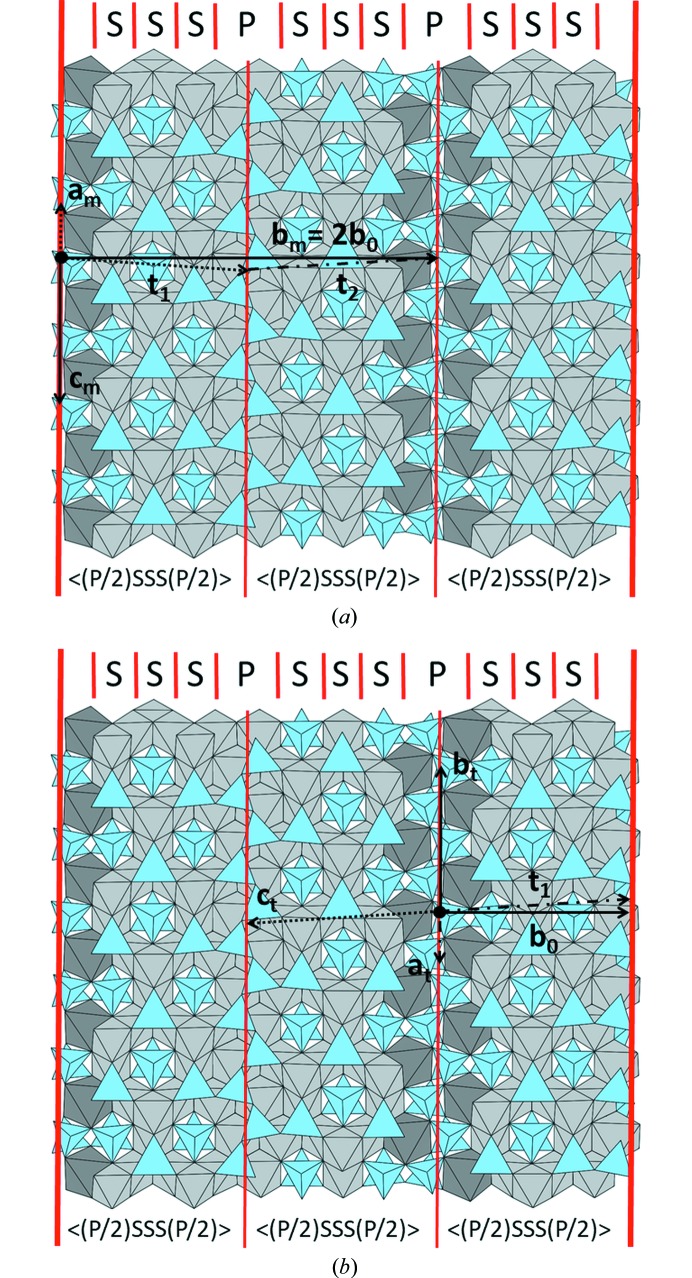
The crystal structures of (*a*) monoclinic and (*b*) triclinic SFCA-III. The sub-division into spinel (S) and pyroxene (P) modules is indicated. Both polytypes correspond to 〈S_3_P〉 sequences of the polysomatic series. Vectors drawn as solid lines lie within the projection plane. Dotted and chain-dotted vectors point downwards and upwards, respectively. For more details concerning the definition of **b_0_, t_1_** and **t_2_** see text.

So far, various stacking sequences 〈S_*m*_P〉 of S- and P-modules have been found. SFCA and SFCA-I, for example, correspond to 〈SP〉 and 〈S_2_P〉 (= 〈SSP〉), respectively. The existence of compounds with *m* > 2 has been challenged by Arakcheeva & Ivanov (1993 ▸). However, the sequence 〈S_3_P〉 (= 〈SSSP〉) containing three consecutive spinel and one pyroxene module was recently found in FCAM-III (Zöll *et al.*, 2018 ▸) and is also a characteristic feature of triclinic as well as monoclinic SFCA-III [see Figs. 8 ▸(*a*) and  ▸8(*b*)]. SFCA-II, on the other hand, corresponds to a member of a more general polysomatic series with the polysomatic formula 〈S_2_PSP〉 (Merlino & Pasero, 1997 ▸).

**Figure 9:**
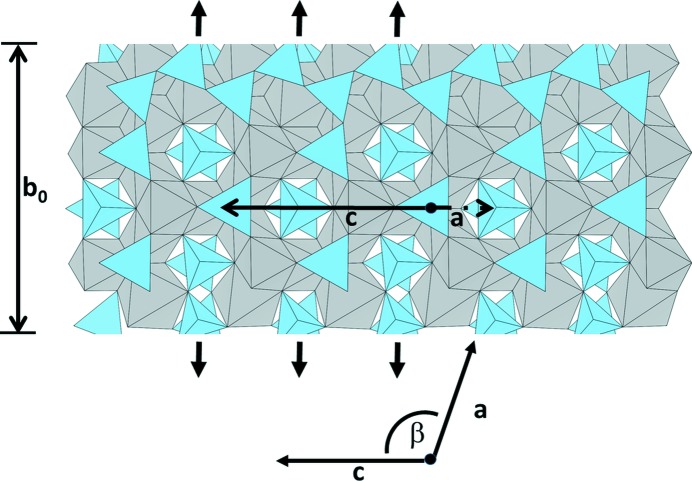
Single OD-layer (〈(P/2)S_3_(P/2)〉) with layer symmetry *P*12/*n*1. The corresponding twofold axes are indicated. b_0_ denotes the thickness of the layer. ***a***_ODL_ and ***c***_ODL_ represent the translation vectors within the layer making an angle β of about 109° with each other (as indicated in the lower part of the figure).

As described by Zvyagin & Merlino (2003 ▸), the members of the 〈S_*m*_P〉 polysomatic series are prone to polytypism. Well known examples from the realm of mineralogy are the sapphirine-1A and sapphirine-2M polytypes (both having 〈SP〉 stacking sequences) (Merlino & Zvyagin, 1998 ▸). According to the detailed theoretical investigation of Zvyagin and Merlino, the tendency to form polytypes can be rationalized on the basis of the theory of OD-structures (see Dornberger-Schiff, 1956 ▸, 1979 ▸; Merlino, 1997 ▸; Ferraris *et al.*, 2008 ▸ and references cited therein) consisting of equivalent layers. In OD structures neighboring layers can be arranged in two or more distinct, but geometrically equivalent ways. The various possible disordered or ordered sequences of the two or more stacking schemes result in a family of disordered or ordered structures (polytypes): pairs of adjacent layers are geometrically equivalent in all the structures of the family. For all 〈S_*m*_P〉 polysomes, OD-layers or stacking domains of the type 〈(P/2)S_*m*_(P/2)〉 can be defined. Depending on whether *m* is even or odd, Zvyagin & Merlino (2003 ▸) demonstrated that a single OD-layer has either layer group symmetry *P*12/*n*1 (*m* = odd) or *P*12/*a*1 (*m* = even). For the present case with *m* = 3 a single OD-layer is given in Fig. 9 ▸, the metric of which is as follows: *a*_ODL_ = 10.33 Å, *c*_ODL_ = 10.43 Å, β = 109.66, *b*_0_ = 13.66 Å, where *b*_0_ corresponds to the thickness of the layer. The corresponding 〈(P/2)S_*m*_(P/2)〉 units in both polytypes have been indicated in Fig. 8 ▸ as well.

However, a full analysis based on OD-theory requires both (i) the symmetry properties of the single OD layer (λ-operators) *and* (ii) the operators that map adjacent layers into each other (σ-operators). The σ-operators for all polysomes independent of their even or odd parity are glide planes perpendicular to the layers with a translational component of ±**c**/4 (denoted *c*_1/2_ or *c*_−1/2_) and twofold axes with a translational component corresponding to the thickness of the layer *b*_0_ (denoted 2_2_) (see Zvyagin & Merlino, 2003 ▸). The full symbol for the resulting OD groupoid family for a polysome with odd parity is
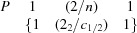



This concise two-line symbol combines the requested information about the layer symmetry and the σ-operators. From a structural point of view, the OD groupoid family represents the whole set of potentially ordered (polytypes) or disordered structures depending on the ordered or disordered sequence of the σ-operators. Though the number of stacking possibilities is infinite, usually only a small number is important, amongst which the maximum degree of order (or MDO) structures stand out (Fichtner, 1988 ▸). They represent those structures in which not only pairs, but also triples, quadruples,…, *n*-tuples of consecutive layers are geometrically equivalent (Dornberger-Schiff, 1982 ▸; Ferraris *et al.*, 2008 ▸). For a *m* = 3 member such as SFCA-III two different MDO structures exist: MDO_1_ corresponds to a strictly uniform 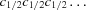 sequence, whereas MDO_2_ is characterized by an alternating 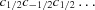 succession. The 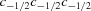 sequence represents no principally new MDO polytype but a twinned structure of MDO_1_. The resulting space group symmetry of MDO_1_ is 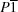. The relationships between its triclinic lattice vectors and those of defining the OD-layer are as follows:
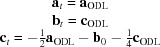



For MDO_2_, the 2_2_-operator has a continuation in adjacent OD-layers and, therefore, transforms into a global symmetry element of the whole crystal structure, *i.e.* forming a 2_1_-screw axis. The space group of the monoclinic MDO_2_ polytype is *P*2_1_/*n* (Zvyagin & Merlino, 2003 ▸) with the following relations between the basis vector of the monoclinic structure and the OD-layer:
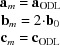



Notably, it can be shown that the application of the c_1/2_ and the c_-1/2_ operation is equivalent to a translation of adjacent OD-layers by vectors **t**_1_ = ½**a**_ODL_ + **b**_0_ + ¼**c**_ODL_ and **t_2_** = –½**a**_ODL_ + **b**_0_ – ¼**c**_ODL_, respectively. Therefore, one could describe the differences between the MDO_1_- and MDO_2_-polytypes alternatively by different shift sequences of subsequent layers according to either **t**_1_ **t**_1_ **t**_1_… (for MDO_1_) or **t**_1_ **t**_2_ **t**_1_… (for MDO_2_) (see Fig. 8 ▸). In summary one can say that the theoretical predictions of Zvyagin & Merlino (2003 ▸) concerning polytypism in the 〈S_*m*_P〉 polysomatic series of the sapphirine-aenigmatite group are perfectly fulfilled for the two modifications observed for SFCA-III. They simply represent the two possible MDO structures. Following the recommendation of the International Union of Crystallography (Guinier *et al.*, 1984 ▸) MDO_1_ and MDO_2_ of SFCA-III could be also called SFCA-III-1A and SFCA-III-2M, respectively.

Furthermore, we would like to comment on the specific sample ‘crystal B’ that was used for structure determination. As mentioned above this multi-crystal contained an oriented intergrowth of two SFCA-III modifications which turned out to be two different polytypes. This phenomenon has been already observed in the literature for other compounds and is known as *allotwinning* (Nespolo *et al.*, 1999 ▸). In allotwins, different polytypes grow together epitactically to form domains large enough to produce sharp Bragg peaks in a single-crystal diffraction study. Allotwins have been described in quite a number of chemically different materials including molecular crystals (Strand *et al.*, 2016 ▸; Kautny *et al.*, 2017 ▸), the mica group of minerals (Nespolo *et al.*, 1999 ▸) or synthetic inorganic phases such as KCa_3_Te_5_O_12_Cl_3_ (Larvor *et al.*, 2018 ▸) and KAgCO_3_ (Hans *et al.*, 2015 ▸) – to mention just a few.

Polytypism – which is a pre-requisite for allotwinning – strongly depends on the crystallization conditions (Ďurovič & Weiss, 1986 ▸). For a given set of crystallization defining parameters such as temperature or chemical composition, for example, either one or another polytype should be energetically preferred. Due to comparatively small structural differences between different polytypes small fluctuations within the temperature and compositional parameter landscape may trigger changes in stability, favoring the occurrence of several more or less well ordered polytypes which may show allotwinning. For the SFCA-III polytypes, the individual stability conditions have not been explored yet. According to the EPMA measurements, the SFCA-III containing sample prepared at 1250°C had a homogeneous composition – at least within the analytical resolution of the method. This may indicate that a variation in chemical composition is not the primary cause inducing the simultaneous presence of two polytypes.

Only very recently Mumme & Gable (2018 ▸) reported the existence of a new monoclinic polymorph of SFCA-II. Similar to SFCA-III, the already known triclinic and the novel monoclinic form of SFCA-II represent two different polytypes, though the refinement of the monoclinic variety already showed a pronounced structural disorder within the winged octahedra of layer type 1. Due to their more complex 〈S_2_PSP〉 stacking sequence both polytypes are no direct members of the abovementioned 〈S_*m*_P〉 polysomatic series. Notably, the authors also described a simultaneous presence of both polytypes in their sample used for the diffraction studies. The volume fraction of the triclinic form, however, was only about 2%. According to Mumme & Gable (2018 ▸) there is a difference in the Fe^2+^ content of both polytypes with the monoclinic form being the more reduced one containing significant Fe^2+^.

To the best of our knowledge, effects of polytypism have not been considered for SFCA-samples related to iron-ore sintering so far and Rietveld refinements used for quantitative phase analysis of the sinter products were based on the well established structure models for the triclinic forms of SFCA and SFCA-I, respectively. From the results of the present investigation on SFCA-III and the previous theoretical studies on polytypism among the *M*_14+6*n*_O_20+8*n*_ polysomatic series (Zvyagin & Merlino, 2003 ▸), it can be concluded that this phenomenon may be much more common and could be probably also encountered when dealing with SFCA’s in real sinters. The industrial sinter-process conditions are significantly ‘harsher’ and farther away from equilibrium then the standard laboratory experiments. Much shorter reaction times as well as much larger temperature gradients and variations in oxygen fugacity within the flame front should facilitate the formation of different polytypes upon crystallization. Therefore, it would be worthwhile to study the crystallography of the bonding-phases in iron-ore sinters in more detail. Single-crystal diffraction would not be the method of choice for this purpose due to small crystallite sizes and the multiphase character of the sintered iron ore including hematite, magnetite and dicalcium silicate, for example. TEM studies, however, could be extremely helpful in detecting the presence of different members of the SFCA family as well as deciphering their intergrowth features. Furthermore, it cannot be excluded that even completely new, more general 〈S_*m*_P_o_〉 stacking sequences may be found. The sequence 〈SP_2_〉, for example, has been observed in the mineral surinamite (Merlino & Pasero, 1997 ▸).

These results may eventually also lead to better fits of powder XRD data and, therefore, improved quantification of SFCA’s in iron-ore sinters by the Rietveld method. So far, the usage of this powerful technique was hindered to some extent by the limited applicability of the published crystal structure models (deVilliers & Verryn, 2007 ▸).

In summary one can say, that the different members of the SFCA polysomatic series and their complex crystal structures are another example of compounds with large technological and economical significance that are not as well understood as one should expect.

## Supplementary Material

Crystal structure: contains datablock(s) SFCA-III-triclinic, SFCA-III-monoclinic. DOI: 10.1107/S2052520619014380/dk5093sup1.cif

Structure factors: contains datablock(s) SFCA-III-triclinic. DOI: 10.1107/S2052520619014380/dk5093SFCA-III-triclinicsup2.hkl

Note about the supporting files. DOI: 10.1107/S2052520619014380/dk5093sup3.txt

CCDC references: 1960602, 1960603

## Figures and Tables

**Table 1:** Experimental details

Crystal data	
Temperature (°C)	22
Radiation type	Synchrotron, λ = 0.72931 Å
μ (mm^−1^)	9.16
Crystal size (mm)	0.02 × 0.02 × 0.03
	
Data collection	
Diffractometer	Aerotech
Absorption correction	Multi scan
*T*_min_, *T*_max_	0.595, 1
No. of reflections	39 442 (overlapping and non-overlapping)
(sin θ/λ)_max_ (Å^−1^)	0.761
	
Refinement	
*R*[*F* ^2^ > 2σ(*F* ^2^)], *wR*(*F* ^2^), *S*	0.037, 0.056, 1.58
No. of independent observed reflections	14 341
No. of parameters	1171

**Table 2:** Structural data for both polytypes

	MDO-1 polytype	MDO-2 polytype
Crystal data		
Chemical formula	Al_9.12_Ca_5.98_Fe_30.7_O_72_Mg_5.34_Si_0.86_	
*M*_r_	3506.1	
Crystal system	Triclinic	Monoclinic
Space group	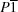	*P*2_1_/*n*
*a*, *b*, *c* (Å)	10.3279 (2), 10.4340 (2), 14.3794 (2)	10.3277 (2), 27.0134 (4), 10.4344 (2)
α, β, γ (°)	93.4888 (12), 107.3209 (14), 109.6626 (14)	90, 109.668 (2), 90
*V* (Å^3^)	1370.49 (5)	2741.22 (9)
*Z*	1	2
		
Refinement		
Δρ_max_,Δρ_min_ (e Å^−3^)	1.11, −1.53	1.09, −0.81
Volume fraction (%)	37.5 (7)	62.5 (7)

**Table 3:** Summary of the EPMA analyses of the phases observed in the synthesis experiments between 1100 and 1300°C The values correspond to the averages of at least ten point analyses. Standard deviations are given in parenthesis. Percentages refer to wt%.

Temperature (°C)	Phases
1100	Quartz
	Hematite
	Melilite (Ca_1.86 (3)_Mg_0.07 (2)_Fe_0.7 (1)_Al_1.34 (4)_Si_1.00 (2)_O_7_)
	SFCA-I (Ca_2.5 (2)_Mg_1.7 (5)_Fe_13.0 (9)_Al_2.5 (1)_Si_0.09 (2)_O_28_)
	FCAM-III (Ca_0.67 (4)_Mg_7.6 (1)_Fe_16.0 (4)_Al_2.5 (3)_O_36_)
1200	Melilite (Ca_1.97 (2)_Mg_0.02 (1)_Fe_0.43 (6)_Al_1.59 (6)_Si_0.99 (2)_O_7_)
	SFCA-III (Ca_3.2 (2)_Mg_2.5 (4)_Fe_15.3 (1)_Al_4.5 (2)_Si_0.37 (9)_O_36_)
1250	SFCA-III (Ca_2.99 (3)_Mg_2.67 (4)_Fe_15.35 (5)_Al_4.56 (4)_Si_0.43 (3)_O_36_)
	Vug filling (CaO: 34.2%, MgO: 0.2%, Fe_2_O_3_: 50.9%, Al_2_O_3_: 8.5%, SiO_2_: 6.7%)
1300	SFCA-I† (Ca_2.09 (3)_Mg_2.05 (3)_Fe_11.44 (2)_Al_4.05 (3)_Si_0.31 (3)_O_28_)
	SFCA-I‡ (Ca_2.9 (3)_Mg_1.4 (3)_Fe_13.1 (2)_Al_2.4 (2)_Si_0.27 (4)_O_28_)
	SFCA-III (Ca_0.67 (2)_Mg_5.22 (4)_Fe_16.97 (2)_Al_3.09 (2)_O_36_)
	Glass matrix (CaO: 30.9%, MgO: 0.1%, Fe_2_O_3_: 55.9%, Al_2_O_3_: 7.5%, SiO_2_: 5.1%)

† Core

‡ Rim
